# The Anti-Inflammatory Effects and Molecular Mechanism of *Citri Reticulatae Pericarpium* Essential Oil: A Combined GC-MS and Network Pharmacology Study

**DOI:** 10.3390/foods14091455

**Published:** 2025-04-23

**Authors:** Junmei Pu, Jiabao Cui, Hui Yang, Jianxin Cao, Shanshan Xiao, Guiguang Cheng

**Affiliations:** 1 Faculty of Food Science and Engineering, Kunming University of Science and Technology, Kunming 650500, China; 2 Yunnan Key Laboratory of Plateau Food Advanced Manufacturing, Kunming 650500, China; 3 Yunnan International Joint Laboratory of Green Food Processing, Kunming 650500, China

**Keywords:** *Citri Reticulatae Pericarpium* essential oil, anti-inflammation, LPS-RAW 264.7 cells, molecular docking

## Abstract

This study investigated the chemical composition and anti-inflammatory effects of essential oils extracted from *Citrus aurantium* flower, *Citrus sinensis*, Brazilian *Citrus sinensis*, *Citrus limon*, *Citrus bergamia*, and *Citri Reticulatae Pericarpium* using steam distillation and gas chromatography-mass spectrometry (GC-MS). Their anti-inflammatory activities were assessed in LPS-stimulated RAW 264.7 cells. Among them, *Citri Reticulatae Pericarpium* essential oil (CRPEO) exhibited the most potent anti-inflammatory effects, with D-Limonene (76.51%), α-Pinene (2.68%), and Linalool (2.11%) as its primary constituents. The CCK-8 assay showed that the essential oil exhibited no cytotoxicity on HaCaT cells at a concentration of 50 μg/mL. CRPEO significantly preserved cell viability and reduced the production of pro-inflammatory mediators, including tumor necrosis factor (TNF)-α, interleukin (IL)-6, IL-1β, and nitric oxide (NO). Gene expression analysis via RT-qPCR further confirmed the downregulation of TNF-α, IL-6, IL-1β, and inducible nitric oxide synthase (iNOS) at the mRNA level. Network pharmacology and molecular docking studies were employed to identify α-Bulnesene as a key bioactive component of CRPEO and revealed that its principal target is the NLR Family Pyrin Domain-Containing 3 (NLRP3) inflammasome. These findings highlight the strong anti-inflammatory potential of CRPEO and suggest its promising therapeutic application for inflammation-related conditions.

## 1. Introduction

Inflammation is a complex and protective biological response initiated by harmful stimuli, such as pathogens, damaged cells, or irritants. It plays a crucial role in defending the body against infections and injuries by initiating repair processes and activating immune responses [1]. However, when inflammation becomes excessive or chronic, it can lead to the development of a variety of diseases, including rheumatoid arthritis, cardiovascular disorders, and neurodegenerative conditions such as Alzheimer’s disease [2]. Chronic inflammation is often characterized by persistent activation of inflammatory mediators, which can cause tissue damage and contribute to disease progression. While synthetic anti-inflammatory drugs, such as nonsteroidal anti-inflammatory drugs (NSAIDs) and corticosteroids, have been widely used to manage these conditions, their long-term use is frequently associated with a range of adverse side effects, including gastrointestinal issues, renal toxicity, and an increased risk of cardiovascular events [3]. These limitations have raised concerns about the safety of conventional treatments and have spurred the search for safer, more effective alternatives.

Ethnopharmacology has long studied the use of traditional medicines, particularly the importance of plant-based remedies in treating diseases. Traditional medicine systems worldwide have recognized the vast potential of plant-derived natural products in combating inflammation, infections, and other health issues. Through the identification of these plants and their chemical constituents, modern science has gradually validated the bioactivities of many traditional plant medicines, particularly in the field of anti-inflammatory applications. In this context, essential oils, derived from aromatic plants, have gained significant attention due to their diverse bioactive properties, including anti-inflammatory, antioxidant, and antimicrobial activities [4]. Among these, essential oils from the *Rutaceae* family, particularly those derived from citrus species, have shown promising therapeutic potential as natural anti-inflammatory agents [5].

The *Rutaceae* family encompasses a wide variety of plants, including *Citrus aurantium* (flower), *Citrus sinensis*, *Citrus limon*, *Citrus bergamia*, and *Citri Reticulatae Pericarpium*, among others [6]. Essential oils are aromatic secondary metabolites found within these plants, consisting of complex mixtures of bioactive compounds [7]. Essential oils from *Rutaceae* plants are widely utilized in traditional medicinal products, with primary bioactivities including anti-inflammatory, antimicrobial, antioxidant, and insecticidal properties. The biological activities of different *Rutaceae* plants exhibit significant variations due to substantial differences in the chemical composition of their essential oils, which are influenced by multiple factors: geographical origin, harvest time, and specific plant parts utilized (e.g., leaves, stems, flowers, fruits, and roots) [8].

Studies have shown that *Ruta chalepensis* L. essential oil from Algeria significantly reduced carrageenan—induced paw edema in mice, comparable to diclofenac, possibly via the inhibition of inflammatory mediators such as serotonin, prostaglandins, and histamine [9]. However, no studies have directly correlated the chemical composition of essential oil with its anti-inflammatory activity. Given the known antioxidant and free radical scavenging potential of *Rutaceae* essential oils, further research may clarify their mechanisms and therapeutic potential in inflammation management [8]. Among these, *Citri Reticulatae Pericarpium* essential oil (CRPEO) stands out due to its traditional use in Chinese medicine for treating respiratory and digestive ailments, as well as its high content of D-Limonene and other bioactive terpenes, which may contribute to its unique therapeutic potential.

The volatile components of *Rutaceae* essential oils mainly consist of monoterpenes (such as D-Limonene) and sesquiterpenes, as well as their oxygenated derivatives, such as aldehydes (e.g., citral), ketones, acids, alcohols (e.g., linalool), and esters [10]. Notably, D-Limonene is the most abundant component in each of these essential oils [11]. D-Limonene has been extensively documented in the literature for its diverse biological activities, including antioxidant, antidiabetic, anti-cancer, anti-inflammatory, and cardioprotective effects [12]. The bioactivity of essential oils is inherently tied to their chemical composition [13]. Given the intricate and diverse nature of essential oil constituents, the identification of bioactive compounds and elucidation of their mechanisms of action have become a central focus in modern phytopharmacological research.

In this study, we aimed to investigate the anti-inflammatory potential of essential oils extracted from six *Rutaceae* plants—*Citrus aurantium* flower, *Citrus sinensis*, Brazilian *Citrus sinensis*, *Citrus limon*, *Citrus bergamia*, and *Citri Reticulatae Pericarpium*—by elucidating their chemical compositions and evaluating their biological activities in vitro. The objective is to identify key bioactive constituents and explore their potential mechanisms of action, particularly their interactions with inflammatory targets. This work seeks to provide a scientific basis for the potential therapeutic application of CRPEO in managing inflammatory conditions, thereby contributing to the broader search for safe and effective natural alternatives to conventional anti-inflammatory drugs.

## 2. Materials and Methods

### 2.1. Essential Oils

The plant essential oils were provided by Dr. Dongbao Hu (Yuxi Normal University). Fresh peels or flowers were collected at Yuxi, Yunnan Province, China (23°19′ N, 101°16′ E). The picking takes place from the end of September to the end of October, a period when the climate is relatively mild, and precipitation gradually decreases while remaining relatively humid. Essential oils were extracted using steam distillation. Briefly, 100 g of dried peel was placed in a distillation apparatus, and steam was passed through the plant material for 5 h. The extracted essential oils were dried over anhydrous sodium sulfate and stored at 4 °C until further analysis [14].

### 2.2. Cell Culture

RAW 264.7 macrophage cells and human epidermal keratinocytes (HaCaT) cells were cultured in Dulbecco’s modified Eagle medium (DMEM) supplemented with 10% fetal bovine serum (FBS) and 1% penicillin-streptomycin at 37 °C in a 5% CO_2_; atmosphere. The RAW 264.7 murine macrophage cell line was provided by the Kunming Institute of Zoology, Chinese Academy of Sciences (Kunming, China). The HaCaT cell line was purchased from the cell bank of the Shanghai Institute of Biochemistry and Cell Biology, Chinese Academy of Sciences (Shanghai, China), and DMEM and FBS were obtained from Gibco (Grand Island, NY, USA).

### 2.3. LPS-Induced Inflammatory Response in RAW 264.7 Cells

Cells were seeded in 96-well plates at a density of 1 × 10^4^ cells/well and allowed to adhere overnight. The cells were then treated with 1 μg/mL lipopolysaccharide (LPS) to induce inflammation [15]. To evaluate the anti-inflammatory effects, culture medium was replaced with serum-free medium containing LPS and various doses of the essential oils (12.5, 25, and 50 μg/mL), and the plate was cultured for another 24 h under the same conditions. LPS was purchased from Sigma-Aldrich (Shanghai, China).

### 2.4. Cell Viability Assay (CCK-8)

Cell viability was assessed using the Cell Counting Kit-8 (CCK-8) assay (Biosharp, Beijing, China). In brief, HaCaT cells (2 × 10^4^ cells/well) were cultured in 96-well plates for 24 h and then treated with various concentrations of essential oils for 20 h. RAW 264.7 macrophage cells were treated with LPS. Then, 20 μL of CCK-8 solution was added to each well, and the plates were incubated for 4 h at 37 °C. The absorbance was measured at 450 nm using a microplate reader. The cell viability was calculated as a percentage relative to the control group (untreated cells).

### 2.5. Measurement of Inflammatory Factors

The levels of inflammatory factors TNF-α, IL-6, and IL-1β in the cell culture supernatants were measured using enzyme-linked immunosorbent assay (ELISA) kits according to the manufacturer’s instructions. The ELISA kits were purchased from Meimian Industrial (Nanjing, China). The NO detection kit detected the NO content in cell supernatants, and was provided by Nanjing Jiancheng Bioengineering Institute (Nanjing, China). Briefly, the supernatants were collected after 24 h of LPS treatment, and the concentrations of TNF-α, IL-6, IL-1β, and NO were determined using corresponding assay kits. The absorbance was measured at 450 nm, and the concentrations were calculated using standard curves.

### 2.6. Real-Time Quantitative PCR (RT-qPCR) Analyses

Total RNA was isolated from RAW 264.7 cells using TRIzol reagent. cDNA was generated using a reverse transcription kit obtained from Takara Bio Inc. (Beijing, China). Quantitative real-time PCR (RT-qPCR) was conducted using SYBR Green Master Mix on a QuantStudio 5 Real-Time PCR System (Accurate Biotechnology Co., Ltd., Changsha, China). The expression levels of TNF-α, IL-6, IL-1β, and inducible nitric oxide synthase (iNOS) mRNA were measured using gene-specific primers. The 2^−ΔΔCt^ method was employed to calculate relative mRNA expression levels, with GAPDH serving as the internal reference. The sequences of the primers used are listed in Table 1.

### 2.7. Gas Chromatography-Mass Spectrometry (GCMS) Analysis

The chemical composition of the essential oils was analyzed using GCMS. The analysis was performed on an Agilent 7890 B gas chromatograph (Agilent Technologies, Palo Alto, CA, USA) coupled with an Agilent 7000 C mass spectrometer (Agilent, Santa Clara, CA, USA). A DB-5 capillary column (30 m × 0.25 mm, 0.25 μm) was used. The oven temperature was programmed as follows: initial temperature of 45 °Cheld for 3 min, then increased to 230 °C at a rate of 8 °C/min, and held for 10 min. Helium was used as the carrier gas at a flow rate of 1.0 mL/min. The mass spectrometer was operated in electron ionization (EI) mode at 70 eV, with a scan range of 35–500 *m*/*z*. A series of n-alkane standards (C6–C26) were analyzed using identical chromatographic conditions to determine retention indices (RIs) for all observed peaks. The components were identified by comparing their mass spectra with those in the NIST 14 library [16].

### 2.8. Network Pharmacology Analysis

To investigate the potential anti-inflammatory mechanisms of CRPEO, a network pharmacology strategy was utilized. The chemical constituents of CRPEO were characterized through GCMS analysis, and their potential targets were predicted using the SwissTargetPrediction (STP) database. Inflammation-associated targets were extracted from the DisGeNET database. A protein–protein interaction (PPI) network was generated using the STRING database, and the “component–target pathway” network was visualized with Cytoscape software (version 3.10.0). Gene Ontology (GO) and Kyoto Encyclopedia of Genes and Genomes (KEGG) pathway enrichment analyses were conducted to elucidate the key pathways associated with the anti-inflammatory effects [17].

### 2.9. Molecular Docking

Molecular docking was conducted to explore the interactions between the primary active compound and its potential target. The three-dimensional (3D) structure of the compound was retrieved from the PubChem database, while the crystal structure of the target protein was acquired from the Protein Data Bank (PDB). To enable efficient identification of the binding sites, AutoDock Tools were employed to define the dimensions of the grid box, which fully encompassed the entire protein structure. The grid box was configured with dimensions of X = 40, Y = 40, Z = 40, and centered at coordinates X = −30.16, Y = −41.36, Z = −36.77. A grid spacing of 0.375 Å was set, and the exhaustiveness parameter was adjusted to 10. Subsequently, the Lamarckian genetic algorithm, integrated within AutoDock Vina (version 1.2.7), was utilized to systematically analyze the docking interactions between the compounds and the receptor, and the binding affinity along with interaction patterns were analyzed using PyMOL software (version 2.6). The compatibility between the active compound and the target protein was evaluated based on the docking score. Typically, a binding energy lower than −4.25 kcal/mol indicates some degree of binding activity between the ligand and the receptor; a value below −5.0 kcal/mol suggests good binding activity, and a value less than −7.25 kcal/mol signifies strong binding activity [17].

### 2.10. Molecular Dynamics (MD) Simulation

The structure of NLRP3 was obtained from the Protein date bank (PBD). The molecular structures of the α-Bulnesene was obtained from PubChem, and the CID number is 94275. All heteroatoms and water molecules were removed using PyMOL software (version 2.6). We set NLRP3 as receptors, and α-Bulnesene as ligands. We used the prepare_ligand and prepare_receptor modules of ADFR to process proteins and small molecules and prepare them in pdbqt format. Finally, global molecular docking was performed using Autodock Vina (version 1.2.7).

Molecular dynamics simulations of α-Bulnesene and NLRP3 were performed using Gromacs software (2024.3). First, the Protoss tool was used to correctly protonate small molecules, and ACPYPE was used to generate topology files for small molecules with BCC charges and the GAFF2 force field. For proteins, we used pdb2gmx to generate topology files, adopting the Amber14SB force field and the SPC/E water model. We combined the topology files of the protein and small molecules, and filled the system with water molecules and sodium and chloride ions for charge neutralization. The system was subjected to energy minimization using the conjugate gradient method, followed by a 100 ps pre-equilibration of the system at 293.15 K, and then a 100 ns molecular dynamics simulation was carried out.

Once the MD simulation was completed, it was necessary to analyze the crucial information related to molecular conformation, structure, and energy. Specifically, the root mean square deviation (RMSD) and radius of gyration (Rg) were calculated to evaluate the convergence and expansion of the molecular structure.

### 2.11. Statistical Analysis

All experiments were conducted in triplicate, and the results are presented as mean ± standard deviation (SD). Before conducting the ANOVA analysis, we performed tests for normality and homogeneity of variances. The results showed that all data across different groups followed a normal distribution and exhibited homogeneity of variances. Therefore, we used Tukey’s test to analyze the significance of differences between the treatment groups. GraphPad Prism 9.0 software was utilized for experimental data analysis. Comparative data between groups were analyzed using the *t*-test, while one-way ANOVA was employed to assess comparisons of multiple groups. *p* < 0.05 and *p* < 0.01 were considered significant differences in the experimental data.

## 3. Results and Discussion

### 3.1. Cytotoxicity

**Figure 1:**
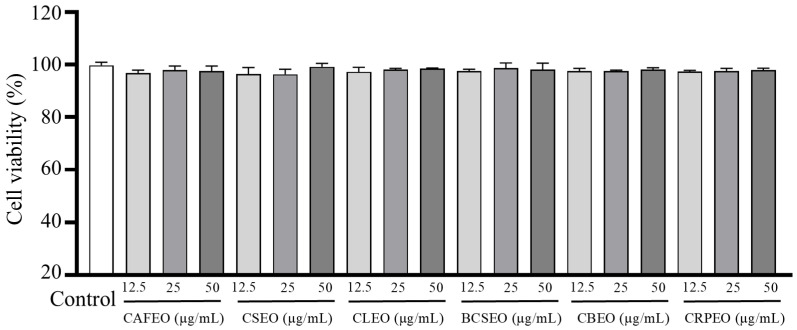
Cytotoxicity of six *Rutaceae* plant essential oils on HaCaT cells. Data were expressed as mean ± SD. Statistical analysis was performed by one-way analysis of variance with Tukey’s multiple comparisons test, results showed that six *Rutaceae* plant essential oils exhibited no significant cytotoxicity. Comparison with control group: *p* > 0.05.

The CCK-8 assay results indicated that the six *Rutaceae* plant essential oils exhibited no significant cytotoxicity toward HaCaT cells at concentrations of 12.5, 25, and 50 μg/mL (Figure 1). These findings suggest that these essential oils may have good biocompatibility at low concentrations. Previous studies have demonstrated that many plant-derived essential oils possess various biological activities, including antimicrobial, anti-inflammatory, and antioxidant effects. However, cytotoxicity remains a critical factor in assessing the safety and efficacy of these essential oils [18].

### 3.2. LPS-Induced Inflammation in RAW 264.7 Cells

LPS (a type of endotoxin in the outer membrane of Gram-negative bacteria) is the most potent inducer of microbial inflammation, triggering shock from multiple organ failure and cellular damage [19]. When macrophages are activated by LPS, they release various pro-inflammatory cytokines and mediators, leading to numerous inflammation-related diseases. Therefore, the LPS-induced RAW 264.7 cell inflammation model has become a common method for evaluating the anti-inflammatory activity of essential oils [13].

**Figure 2:**
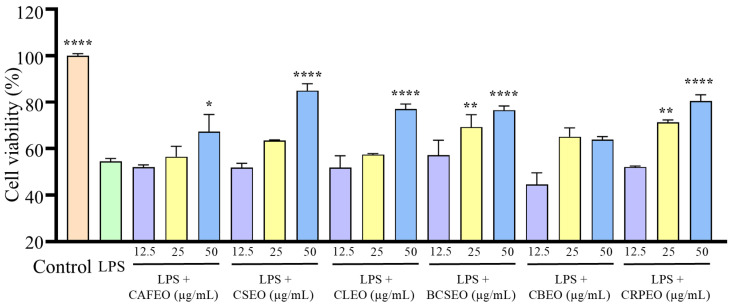
Cytotoxicity of six *Rutaceae* plant essential oils and LPS co-culture with RAW 264.7 cells. Data were expressed as mean ± SD. Statistical analysis was performed by one-way analysis of variance with Tukey’s multiple comparisons test, * *p* < 0.05, ** *p* < 0.01, and **** *p* < 0.0001.

In this study, we evaluated the effects of essential oils on LPS-induced inflammation in RAW 264.7 cells. The CCK-8 assay showed that, compared to the control group, cell viability significantly decreased in the LPS-treated group (Figure 2), which is consistent with previous studies [20]. However, when treated with essential oils, the reduction in cell viability induced by LPS was significantly inhibited, with cell survival rates increasing in a dose-dependent manner, suggesting that the essential oils provided a protective effect against LPS-induced cellular inflammation.

**Figure 3:**
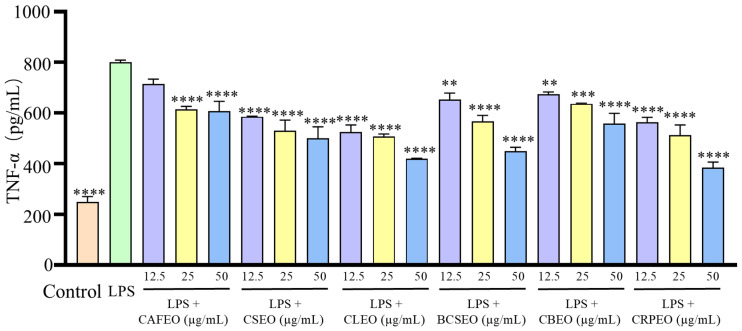
Inhibition effects of six *Rutaceae* plant essential oils on TNF-α production by LPS-stimulated RAW 264.7 cells. Data were expressed as mean ± SD. Statistical analysis was performed by one-way analysis of variance with Tukey’s multiple comparisons test, ** *p* < 0.01, *** *p* < 0.001, and **** *p* < 0.0001.

**Figure 4:**
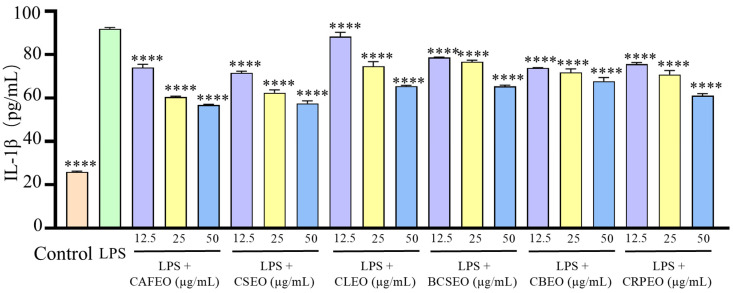
Inhibition effects of six *Rutaceae* plant essential oils on IL-1β production by LPS-stimulated RAW 264.7 cells. Data were expressed as mean ± SD. Statistical analysis was performed by one-way analysis of variance with Tukey’s multiple comparisons test, **** *p* < 0.0001.

**Figure 5:**
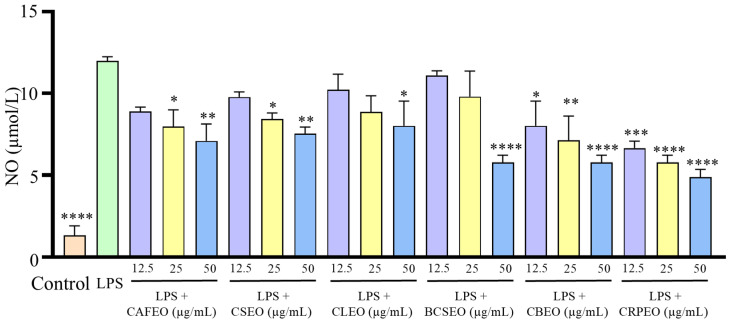
Inhibition effects of six *Rutaceae* plant essential oils on NO production by LPS-stimulated RAW 264.7 cells. Data were expressed as mean ± SD. Statistical analysis was performed by one-way analysis of variance with Tukey’s multiple comparisons test, * *p* < 0.05, ** *p* < 0.01, *** *p* < 0.001, and **** *p* < 0.0001.

**Figure 6:**
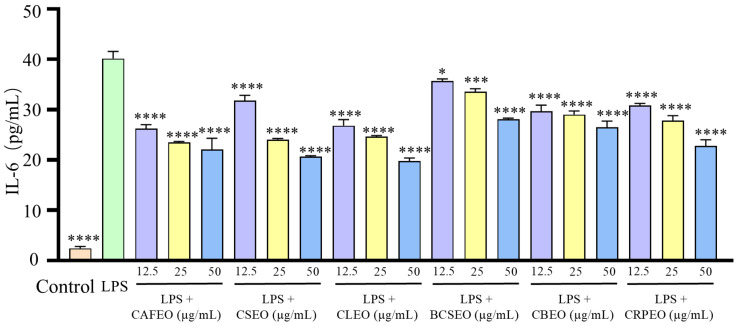
Inhibition effects of six *Rutaceae* plant essential oils on IL-6 production by LPS-stimulated RAW 264.7 cells. Data were expressed as mean ± SD. Statistical analysis was performed by one-way analysis of variance with Tukey’s multiple comparisons test, * *p* < 0.05, *** *p* < 0.001, and **** *p* < 0.0001.

Further analysis of inflammatory factors in the cell supernatant revealed that LPS significantly increased the release of TNF-α (Figure 3), IL-1β (Figure 4), NO (Figure 5), and IL-6 (Figure 6). Compared to the control group, the levels of TNF-α in the model group showed a significant increase from 249.30 ± 21.29 ng/L to 800.51 ± 8.51 ng/L. However, treatment with different essential oils at concentrations of 12.5, 25, and 50 μg/mL effectively inhibited the excessive secretion of inflammatory factors. Specifically, the inhibition rates for TNF-α were 10.71 ± 3.34%, 23.32 ± 2.41%, and 24.10 ± 3.98% in CAFEO; 26.9 ± 1.04%, 33.83 ± 6.03%, and 37.41 ± 6.25% in CSEO; 34.44 ± 2.76%, 36.68 ± 2.00%, and 47.60 ± 0.29% in CLEO; 18.45 ± 2.32%, 29.17 ± 2.17%, and 43.84 ± 1.26% in BCSEO; 15.79 ± 1.96%, 20.50 ± 1.11%, and 30.31 ± 4.31% in CBEO; and 29.55 ± 1.64%, 35.90 ± 5.74%, and 51.94 ± 2.15% in CRPEO.

Similarly, compared to the control group, the levels of IL-Iβ in the model group also significantly increased from 25.94 ± 0.37 ng/L to 91.96 ± 0.56 ng/L. After treatment with different essential oils at concentrations of 12.5, 25, and 50 μg/mL, the inhibition rates for IL-Iβ were 19.50 ± 1.14%, 34.23 ± 0.01%, and 38.28 ± 0.79% in CAFEO; 22.10 ± 0.34%, 32.06 ± 1.85%, and 37.42 ± 0.84% in CSEO; 3.89 ± 2.63%, 18.78 ± 1.75%, and 28.74 ± 0.85% in CLEO; 14.44 ± 0.32%, 16.61 ± 1.33%, and 28.89 ± 1.05% in BCSEO; 19.64 ± 0.70%, 21.95 ± 2.32%, and 26.28 ± 2.29% in CBEO; and 17.76 ± 1.32%, 22.96 ± 2.51%, and 33.51 ± 1.43% in CRPEO.

The levels of NO also showed a significant decrease after treatment with different essential oils at concentrations of 12.5, 25, and 50 μg/mL, with inhibition rates of 25.82 ± 3.89%, 33.52 ± 6.99%, and 40.93 ± 7.38% in CAFEO; 18.41 ± 4.27%, 29.67 ± 1.55%, and 37.09 ± 1.94% in CSEO; 14.56 ± 9.71%, 26.10 ± 6.60%, and 32.97 ± 13.99% in CLEO; 7.42 ± 0.39%, 18.13 ± 14.76%, and 51.92 ± 2.72% in BCSEO; 32.97 ± 13.99%, 40.38 ± 13.60%, and 51.92 ± 2.72% in CBEO; and 44.51 ± 2.33%, 51.92 ± 2.72%, and 59.34 ± 3.11% in CRPEO.

The initial content of IL-6 in the control group was 2.41 ± 0.40 ng/L; after treatment with LPS, the secreted amount of IL-6 increased to 40.14 ± 1.41 ng/L. After treatment with different essential oils at concentrations of 12.5, 25, and 50 μg/mL, the inhibition rates for IL-6 were 34.66 ± 0.29%, 41.40 ± 2.58%, and 45.09 ± 3.62% in CAFEO; 20.63 ± 5.33%, 39.99 ± 1.62%, and 48.55 ± 2.33% in CSEO; 33.14 ± 5.40%, 38.54 ± 2.68%, and 50.68 ± 3.26% in CLEO; 11.40 ± 4.16%, 16.41 ± 1.44%, and 29.98 ± 1.97% in BCSEO; 25.98 ± 5.65%, 27.62 ± 4.32%, and 33.96 ± 0.70% in CBEO; and 23.16 ± 3.73%, 30.64 ± 4.98%, and 43.25 ± 1.02% in CRPEO.

The above results indicate that essential oils have a significant inhibitory effect on the release of inflammatory factors. As the concentration of essential oils gradually increases, their inhibitory efficiency on inflammatory factors shows a gradient-enhancing trend, presenting a typical dose–response relationship.

Among the six species of *Rutaceae* plant essential oils, CRPEO demonstrated the most potent anti-inflammatory effects. To further investigate its mechanism, RT-qPCR was performed to measure the mRNA expression levels of IL-6, iNOS, TNF-α, and IL-1β. The results showed that CRPEO significantly downregulated the mRNA expression of inflammatory factors. Compared to the control group, the mRNA level of TNF-α in the model group increased from 0.83 ± 0.14 to 13.27 ± 0.60. However, treatment with CRPEO at concentrations of 12.5, 25, and 50 μg/mL effectively inhibited the mRNA expression of TNF-α, with inhibition rates of 21.93 ± 4.71%, 35.92 ± 10.64%, and 76.86 ± 2.26%, respectively. Similarly, the mRNA level of IL-1β in the model group significantly increased from 0.70 ± 0.26 to 171.55 ± 9.29 compared to the control group. After treatment with CRPEO, the inhibition rates were 23.50 ± 9.31%, 32.31 ± 3.56%, and 53.34 ± 2.26% at the corresponding concentrations. The expression level of iNOS also showed a significant decrease following CRPEO treatment, with inhibition rates of 30.44 ± 17.40%, 45.25 ± 15.12%, and 65.58 ± 11.24%. The initial mRNA level of IL-6 in the control group was 0.57 ± 0.36. After LPS stimulation, it increased dramatically to 5158.85 ± 443.77. Treatment with CRPEO reduced IL-6 expression significantly, with inhibition rates of 67.77 ± 5.27%, 71.11 ± 2.92%, and 73.48 ± 4.61%, respectively.

**Figure 7:**
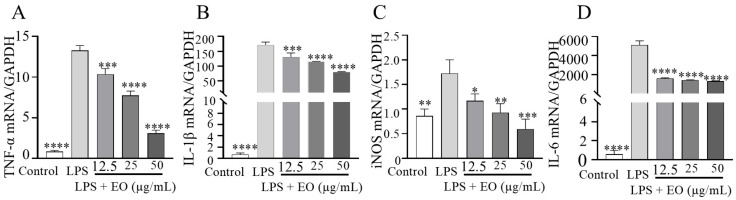
The effects of CRPEO on the mRNA expression of pro-inflammatory cytokines. CRPEO inhibits LPS-induced mRNA levels of TNF-α (**A**), IL-1β (**B**), iNOS (**C**), and IL-6 (**D**) in RAW 264.7 cells. Data were expressed as mean ±SD. Statistical analysis was performed by one-way analysis of variance with Tukey’s multiple comparisons test, * *p* < 0.05, ** *p* < 0.01, *** *p* < 0.001, and **** *p* < 0.0001.

The results showed that CRPEO significantly downregulated the mRNA expression of key inflammatory factors, consistent with the results obtained from the ELISA (Figure 7). These findings suggest that CRPEO may exert its anti-inflammatory effects by modulating crucial inflammatory pathways and targets, laying a theoretical foundation for its potential application in managing inflammation-related conditions. Given the central role of pro-inflammatory cytokines such as TNF-α, IL-1β, and IL-6 in the pathogenesis of common chronic inflammatory diseases—including rheumatoid arthritis, inflammatory bowel disease, and neurodegenerative disorders—our results highlight the potential relevance of CRPEO as a complementary or alternative therapy. This is particularly significant considering the adverse effects associated with the long-term use of conventional anti-inflammatory drugs, such as NSAIDs and corticosteroids [21]. Nevertheless, as in vitro models cannot fully recapitulate the complexity of inflammation in a physiological environment, further in vivo validation is necessary. In vivo studies are crucial to determine bioavailability, metabolic stability, systemic toxicity, and potential synergistic effects among components within a living organism. Moreover, clinical trials are essential to confirm both efficacy and safety in human populations, which cannot be reliably inferred from in vitro data alone.

Considering the complex chemical nature of essential oils and their multi-target interactions, we further conducted integrated network pharmacology and molecular docking analyses to systematically explore the bioactive components and molecular mechanisms responsible for the observed anti-inflammatory effects of CRPEO.

### 3.3. Chemical Components of Rutaceae Essential Oils

The chemical composition of essential oils (EOs) extracted from six species of *Rutaceae* plants was analyzed and identified by GC-MS. A total of 104 compounds were detected across the six essential oils. *Citrus aurantium* flower essential oil (CAFEO) contained 19 compounds, accounting for 98.28% of the total essential oil composition, with the main components being D-Limonene (57.77%), γ-Terpinene (11.29%), and Terpinolene (5.50%), among others (Table A1). These findings are consistent with previous studies that have reported D-Limonene as a dominant compound in neroli oil, and it has also been shown to exhibit significant anti-inflammatory properties [22]. A research study reported that neroli oil, extracted from fresh *Citrus aurantium* L. flowers grown in the Nabeul region of northeastern Tunisia, contained limonene (27.5%) as the major constituent, followed by (E)-nerolidol (17.5%), α-terpineol (14%), and α-terpinyl acetate (11.7%) [23]. We speculate that the differences in composition may be attributed to the influence of the geographical origin.

*Citrus sinensis* essential oil (CSEO) contained 27 compounds, accounting for 90.28% of the total essential oil composition. The primary constituents were D-Limonene (35.06%), Carveol (12.44%), and p-Mentha-2,8-dienol (11.61%) (Table A2). In previous studies, the main components of CSEO were found to be limonene (53.9%), β-pinene (13.1%), and thujene (10.52%), among others [24]. This variation in chemical composition may be attributed to differences in geographic origin, cultivation conditions, harvest time, or extraction methods, highlighting the influence of environmental and processing factors on the phytochemical profile of essential oils.

*Citrus limon* essential oil (CLEO) contained 14 compounds, accounting for 91.78% of the total essential oil composition. The main components were α-Fenchene (30.19%), D-Limonene (21.74%), and Linalyl Formate (15.46%) (Table A3). A study has shown that the main components of CLEO are Z-citral (53.21%), neryl acetate (13.06%), geranyl acetate (10.33%), and limonene (4.23%) [25]. The differences in the chemical composition of CLEO in these two studies may be attributed to factors such as the geographical origin.

Brazilian *Citrus sinensis* essential oil (BCSEO) contained 34 compounds, accounting for 90.77% of the total essential oil composition. The major components were D-Limonene (34.60%), Linalool (7.07%), and α-Terpineol (6.97%) (Table A4). In the study by Padilla-Camberos et al., the BCSEO obtained through hydrodistillation contained D-limonene (82.97%), β-caryophyllene (2.73%), and α-pinene (1.52%), among others. The high content of D-limonene may be related to the essential oil extraction method [26].

*Citrus bergamia* essential oil (CBEO) contained 23 compounds, accounting for 94.03% of the total essential oil composition. The primary constituents were Citronellol (29.56%), Guaia-6,9-Diene (11.52%), and Isomenthone (10.62%) (Table A5). The composition of CBEO, particularly the high levels of Citronellol, distinguishes it from other citrus oils and may contribute to its distinct aroma and therapeutic properties [27]. In the study by Rasheed et al., the main components of CBEO were found to be D-Limonene (23.21%), Linalyl acetate (14.01%), and Linalool (9.96%), among others [28]. From the perspective of composition, this shows a clear difference from our essential oil, which may also explain the differences in their bioactive effects.

CRPEO contained 23 compounds, accounting for 90.36% of the total essential oil composition. The main components were D-Limonene (76.51%), α-Pinene (2.68%), and Linalool (2.11%) (Table 2). The high content of D-Limonene may be the key to the pharmacological effects of CRPEO [29]. Studies have shown that the main components of tangerine peel essential oil produced in Guangzhou include D-limonene (55.85%), γ-terpinene (13.83%), and α-pinene (1.84%). Compared to our results, where D-limonene (76.51%), α-pinene (2.68%), and linalool (2.11%) were the major constituents, there are noticeable differences in both the limonene content and the overall composition. This supports the notion that the geographical origin significantly influences the chemical profile of essential oils [30].

The chemical composition is a critical determinant of the biological and pharmacological effects of essential oils. D-Limonene exerts anti-inflammatory effects by suppressing the release of inflammatory mediators, including TNF-α and IL-6, and regulating oxidative stress [29]. Furthermore, the high concentration of Citronellol (29.56%) in CBEO may contribute to its distinct anti-inflammatory and antioxidant properties [28]. These findings indicate that the chemical composition of essential oils not only governs their aromatic and flavor characteristics but also significantly impacts their biological activities and therapeutic potential. Therefore, an LPS-induced RAW 264.7 cell inflammation model was established to evaluate the anti-inflammatory effects of the essential oils.

### 3.4. “Component–Target Pathway” of the Anti-Inflammatory Effects of Essential Oils

**Figure 8:**
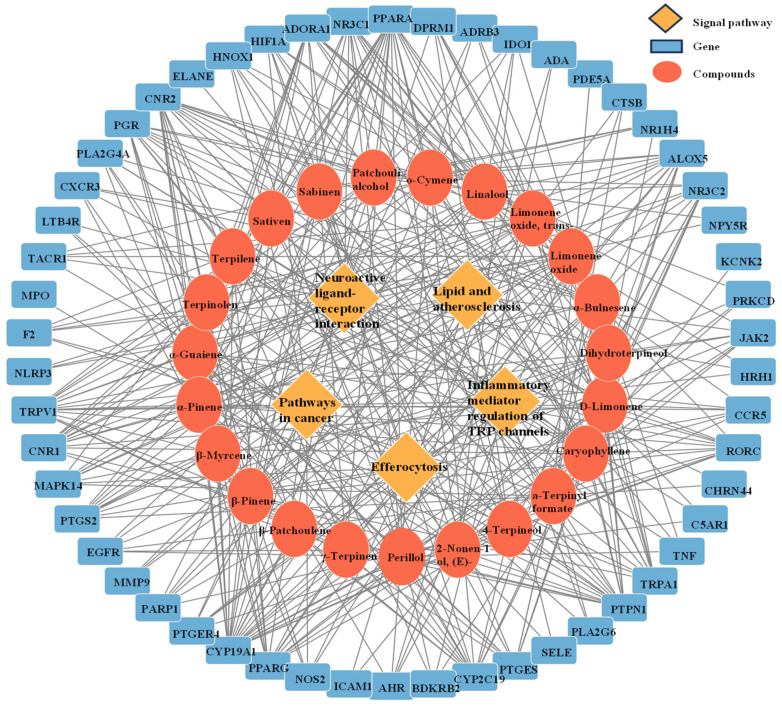
The anti-inflammatory network of the “component–target signaling pathways” regulated by CRPEO. The orange rhomboidal nodes represent pathways; the blue rectangle nodes represent targets; and the round nodes represent components.

Based on network pharmacology approaches, database searches identified 23 components in CRPEO associated with inflammation and 54 corresponding targets, as shown in Figure 8. Among the 23 active components, α-Bulnesene, α-Pinene, Terpinyl formate, and Caryophyllene were connected to 22, 20, 19, and 19 targets, respectively, suggesting that these compounds play a central role in mediating the anti-inflammatory effects of CRPEO.

α-Pinene, a monoterpene, is known for its ability to reduce pro-inflammatory cytokine production and oxidative stress [31]. Caryophyllene, a bicyclic sesquiterpene, acts as a selective agonist of the cannabinoid receptor type 2 (CB2), which is involved in inflammation regulation [32]. Similarly, α-Guaiene, another sesquiterpene, has demonstrated anti-inflammatory effects by inhibiting cyclooxygenase-2 (COX-2) expression [33].

The high number of targets associated with these compounds suggests that they may exert their effects through a synergistic mechanism, targeting multiple pathways simultaneously [34]. Further experimental validation, including in vitro and in vivo studies, is necessary to confirm the specific mechanisms of action and therapeutic potential of these compounds.

**Figure 9:**
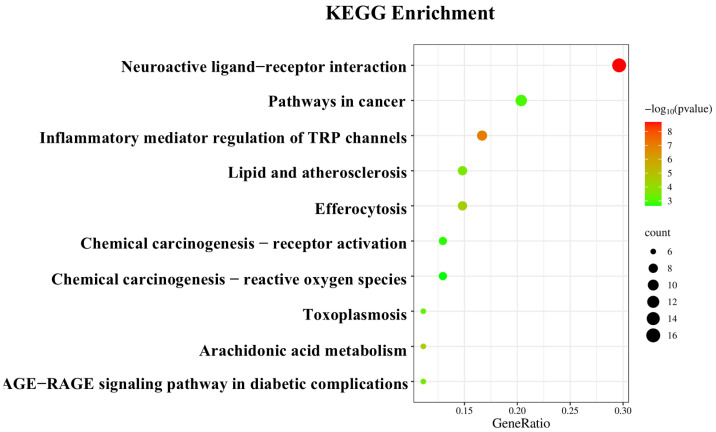
The top 10 KEGG pathways associated with the enrichment of the therapeutic targets.

The KEGG pathway analysis, results are shown in Figure 9. The 59 targets are primarily involved in the following signaling pathways: neuroactive ligand–receptor interaction (16 targets), pathways in cancer (11 targets), inflammatory mediator regulation of TRP channels (9 targets), efferocytosis (8 targets), and lipid and atherosclerosis (8 targets). These findings suggest that the anti-inflammatory effects of CRPEO are mediated through a multi-pathway regulatory mechanism.

The involvement of neuroactive ligand–receptor interaction highlights the potential role of CRPEO in modulating neuroinflammation, which is increasingly recognized as a critical factor in chronic inflammatory diseases [35]. The significant representation of Pathways in cancer suggests that CRPEO may also exert anti-cancer effects by targeting inflammation-related oncogenic pathways, such as NF-κB and PI3K/AKT [36]. These results suggest that essential oils act through multi–component, multi–target, and multi–signaling pathways. Further experimental studies are needed to validate these findings.

### 3.5. GO Enrichment Analysis

**Figure 10:**
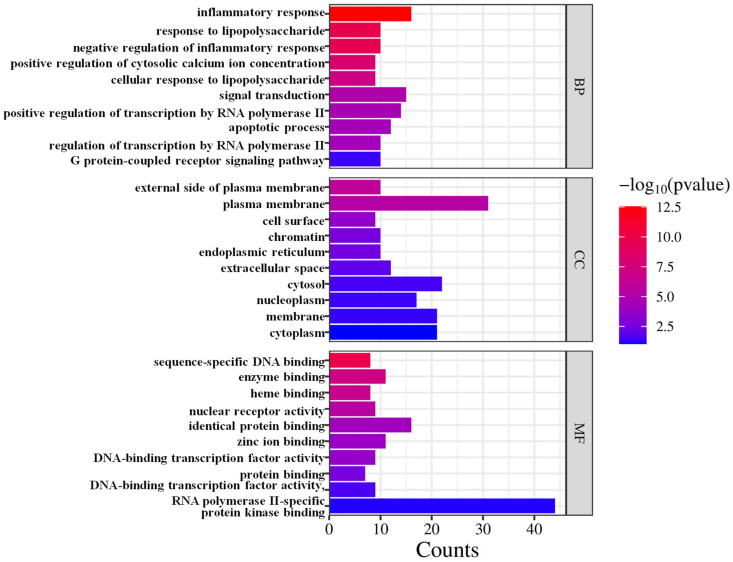
The Gene Ontology (GO) enrichment analysis.

The GO enrichment analysis revealed that the 54 targets regulated by CRPEO, which are associated with its anti-inflammatory effects, are primarily involved in a variety of biological processes and molecular functions, including inflammatory response, signal transduction, positive regulation of transcription by RNA polymerase II, plasma membrane, cytoplasm, cytosol, identical protein binding, zinc ion binding, and enzyme binding (Figure 10).

The enrichment of inflammatory response and signal transduction pathways highlights the central role of CRPEO in modulating key inflammatory signaling cascades, such as NF-κB and MAPK pathways [37]. Additionally, the localization of targets in the plasma membrane, cytoplasm, and cytosol indicates that CRPEO may influence cellular signaling and communication across different cellular compartments. The molecular functions of identical protein binding, zinc ion binding, and enzyme binding further suggest that active components may interact with specific proteins or enzymes to exert their anti-inflammatory effects.

### 3.6. Protein–Protein Interaction (PPI) Network Analysis

**Figure 11:**
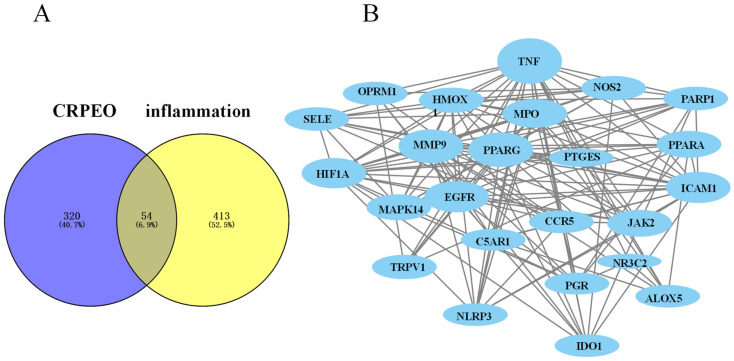
The PPI network analysis. (**A**) The identification of 54 potential therapeutic targets based on the 23 components of CRPEO in the treatment of inflammation. (**B**) The protein–protein interaction network of 54 genes.

The PPI network analysis revealed that the 54 common targets between CRPEO and inflammation-related pathways form a highly interconnected network, with EGFR and JAK2 exhibiting the strongest binding affinity (combined score: 0.986) (Figure 11). This suggests that these interaction pairs may play a critical role in mediating the anti-inflammatory effects of CRPEO. Additionally, STRING analysis identified 24 hub targets with a node degree higher than the average (11.54), including well-known inflammatory regulators such as MAPK14, NOS2, NR3CI, and TNF [38,39,40]. These hub targets are likely to be central to the anti-inflammatory mechanism of CRPEO, as they are involved in key inflammatory processes such as cytokine signaling, oxidative stress, and immune response modulation.

### 3.7. Molecular Docking Analysis

**Figure 12:**
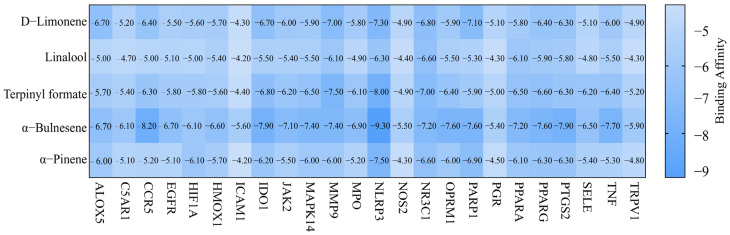
Th heatmap generated to display the docking scores for each component–target pair.

**Figure 13:**
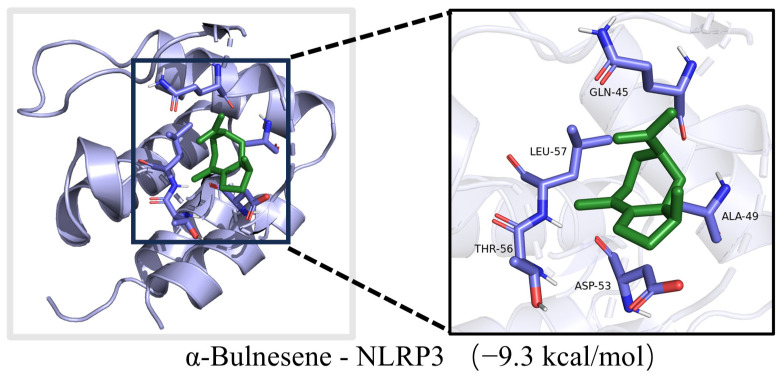
The molecular docking diagram of α-Bulnesene−NLRP3.

The top three components in CRPEO by content are D-Limonene (76.51%), α-Pinene (2.68%), and Linalool (2.11%). Based on the “degree” values from the network pharmacology analysis, the top three components are α-Bulnesene (0.34%), α-Pinene (2.68%), Terpinyl formate (0.28%). Molecular docking was performed between these components and the core inflammation-related targets. The systems with the lowest docking scores for each component–target pair are shown in Figure 12. Notably, 50.83% of the component–target pairs exhibited docking scores lower than 6 kcal/mol, indicating strong binding affinity. The combinations with the lowest docking energies were α-Bulnesene−NLRP3 (Figure 13). These results suggest that the aforementioned compounds and targets are the primary active compounds and key targets responsible for the anti-inflammatory effects of CRPEO.

It is noteworthy that the molecular docking results revealed the combination with the lowest binding energy to be α-Bulnesene−NLRP3, where α-Bulnesene constitutes only 0.34% of the essential oil. This finding suggests that even minor constituents may play a critical role in the observed bioactivity, potentially acting synergistically with major components. Such interactions emphasize the importance of viewing the essential oil as an integrated, multi-component system rather than focusing solely on its predominant compounds. Further experimental validation, particularly through in vivo studies, is necessary to confirm these interactions and assess the pharmacodynamic and pharmacokinetic relevance of both major and minor constituents. These findings underscore the practical value of comprehensive profiling when evaluating the therapeutic potential of complex natural mixtures such as essential oils.

### 3.8. Molecular Dynamics Simulation Analysis

**Figure 14:**
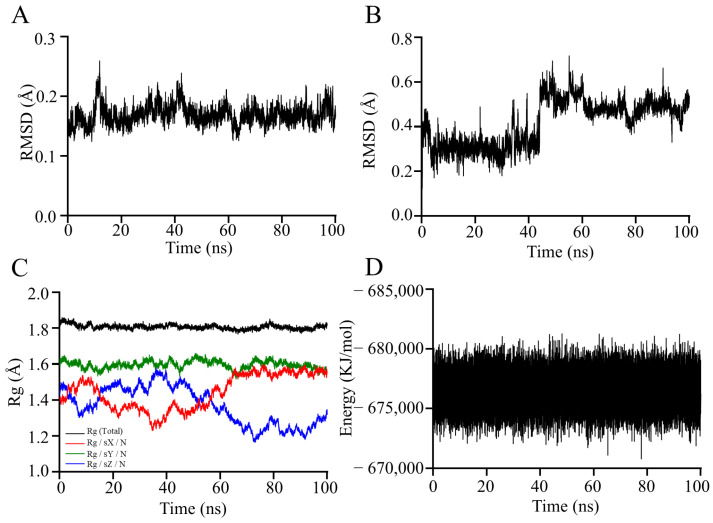
Molecular dynamics analysis. (**A**) RMAD changes in NLRP3. (**B**) RMAD changes in α-Bulnesene. (**C**) The Rg of the complex. (**D**) The total docking value of molecular dynamics simulation.

Molecular dynamics simulations can be used to study the state of molecular motion in real time, which can lead to a better understanding of the structural changes and mechanism of α-Bulnesene in complexation with NLRP3. The RMSD value measures the extent to which an atom has deviated from its initial position, reflecting the convergence and stability of the molecular structure. A higher RMSD value indicates a higher spatial range of motion of the atom in question, suggesting less steric hindrance of the atoms [41]. As shown in Figure 14A,B, the RMSD values of the NLRP3 was 0.16 Å, it indicates that there is no significant conformational change in the protein. The conformation of the α-Bulnesene is in a state of change, indicating that it rotates within the active pocket rather than flying out of it.

The Rg can be used to assess the strength of the molecular structure [42]. As the Rg value decreases, the molecular structure converges and becomes more stable. Conversely, as the Rg value increases, the molecular structure becomes looser or is stretched [43]. Figure 14C shows that the complex formed by the NLRP3 and the α-Bulnesene maintained a stable conformation throughout the simulation. The overall Rg value remained relatively low, indicating a compact structure. However, fluctuations in the Rg values along the X and Z axes were observed, suggesting that the complex underwent local loosening, stretching, or tightening processes. These structural changes can be attributed to the rotation of the α-Bulnesene within the active pocket of the protein, which is consistent with the observed fluctuations in the RMSD of the α-Bulnesene during the docking process. This correlation between the Rg and RMSD values provides further evidence for the dynamic behavior of the α-Bulnesene within the binding site.

In the research of molecular docking and molecular dynamics simulations, the total docking value from molecular dynamics simulations serves as a crucial indicator comprehensively reflecting the interaction between α-Bulnesene and NLRP3. As shown in Figure 14D, the docking value remains stable within the range of −670,758.125 to −681,294.1875 KJ/mol. This result indicates that there is a strong binding force between α-Bulnesene and NLRP3, suggesting that α-Bulnesene is highly likely to bind tightly to NLRP3, thereby exerting an anti-inflammatory effect. Meanwhile, it also reflects that the structure of the complex is relatively stable.

Although the total docking value cannot be completely equated with the actual efficacy of a drug, it is closely related to the binding ability between small molecules and proteins. Generally speaking, molecules with better docking values are more likely to have higher biological activity [44]. Therefore, this total docking value provides important theoretical support for the subsequent in-depth anti-inflammatory experiments of CRPEO.

Although our computational analyses (including molecular docking and molecular dynamics simulations) strongly suggest potential interactions between α-Bulnesene and NLRP3 (Figure 13 and Figure 14), it must be emphasized that these predictions require additional functional experimental validation. It is important to note that these predictions require experimental validation.

## 4. Conclusions

The primary objective of this study was to investigate the anti-inflammatory potential of essential oils extracted from various *Rutaceae* plants, with a particular focus on CRPEO. The findings demonstrate that CRPEO, which contains D-Limonene as its primary component (76.51%), exhibits significant anti-inflammatory effects by inhibiting the production of key inflammatory mediators such as TNF-α, IL-6, IL-1β, and NO. CRPEO also downregulated the mRNA expression of these inflammatory markers, further supporting its anti-inflammatory activity.

Through network pharmacology and molecular docking analyses, α-Bulnesene was identified as the main active compound, and NLRP3 was pinpointed as its key target, illustrating the multi-target and multi-pathway mechanisms behind the anti-inflammatory effects of CRPEO. These results underscore the therapeutic potential of tangerine peel essential oil in managing inflammation-related conditions.

This study provides a scientific basis for the traditional use of CRPEO and highlights its potential as a promising candidate for the development of novel anti-inflammatory therapies. However, further in vivo studies and clinical trials are needed to fully assess its therapeutic efficacy and safety profile.

## Figures and Tables

**Table 1:** Primer sequences used for RT-qPCR analysis.

Gene	Primer Sequences
TNF-α	F: 5′ TTCTGTCTACTGAACTTC3′ R: 5′ CCATAGAACTGATGAGAG3′
IL-6	F: 5′ GCCAGAGTCCTTCAGAGAGA3′ R: 5′ TGGTCCTTAGCCACTCCTTC3′
IL-1β	F: 5′ CAATGGACAGAATATCAAC3′ R: 5′ ACAGGACAGGTATAGATT3′
iNOS	F: 5′ TACGGAAGTCAGAAGATG3′ R: 5′ TAATGGAGGAGTAGTATTGG3′
GAPDH	F: 5′ AGTGGCAAAGTGGAGATT3′ R: 5′ GTGGAGTCATACTGGAACA3′

**Table 2:** Chemical components of CRPEO by GC-MS analysis.

No.	Compound	Content (%)	RI		RT (min)
			Measured	Documented	
1	α-Pinene	2.68	933	931	7.397
2	β-Pinene	0.34	976	975	8.852
3	β-Myrcene	0.81	991	988	9.127
4	2-Nonen-1-ol, (E)-	0.23	1144	1149	9.484
5	o-Cymene	1.3	1023	1018	10.427
6	D-Limonene	76.51	1031	1028	10.622
7	Sabinen	0.32	965	969	10.686
8	γ-Terpinene	1.20	1054	1056	11.601
9	Terpilene	0.16	1019	1016	11.845
10	Terpinolen	0.56	1082	1083	12.586
11	Linalool	2.11	1100	1101	13.036
12	Limonene oxide	0.22	1140	1138	14.3
13	Limonene oxide, trans-	0.25	1137	1139	14.439
14	Perillol	0.34	1291	1294	14.645
15	Dihydroterpineol	0.62	1145	1142	14.904
16	4-Terpineol	0.14	1176	1180	16.03
17	Terpinyl formate	0.28	1181	1183	16.536
18	β-Patchoulene	0.38	1384	1381	23.089
19	Caryophyllene	0.29	1411	1415	24.177
20	α-Guaiene	0.61	1440	1442	24.588
21	Sativene	0.45	1394	1390	25.155
22	α-Bulnesene	0.34	1396	1394	26.666
23	Patchouli alcohol	0.22	1661	1663	31.702
	Total (%)	90.36			

## Data Availability

The original contributions presented in the study are included in the article. Further inquiries can be directed to the corresponding authors.

## Abbreviations

The following abbreviations are used in this manuscript:

GC-MS	gas chromatography-mass spectrometry
TNF-α	tumor necrosis factor (TNF)-α
IL-6	interleukin (IL)-6
NO	nitric oxide
iNOS	inducible nitric oxide synthase
NLRP3	NLR Family Pyrin Domain-Containing 3
HaCaT	human epidermal keratinocytes
DMEM	Dulbecco’s modified Eagle medium
FBS	fetal bovine serum
LPS	lipopolysaccharide
CCK-8	Cell Counting Kit-8
ELISA	enzyme-linked immunosorbent assay
RT-qPCR	real-time quantitative PCR
STP	SwissTargetPrediction
PPI	protein–protein interaction
GO	Gene Ontology
KEGG	Kyoto Encyclopedia of Genes and Genomes
3D	three-dimensional
PDB	Protein Data Bank
SD	standard deviation
EOs	essential oils
CAFEO	*Citrus aurantium* flower essential oil
CSEO	*Citrus sinensis* essential oil
CLEO	*Citrus limon* essential oil
BCSEO	Brazilian *Citrus sinensis* essential oil
CBEO	Citrus bergamia essential oil
CRPEO	*Citri Reticulatae Pericarpium* essential oil
CB2	cannabinoid receptor type 2
COX-2	cyclooxygenase-2

## Appendix A

**Table A1:** Chemical components of CAFEO by GC-MS analysis.

No.	Compound	Content (%)	RI		RT (min)
			Measured	Documented	
1	Pseudolimonene	3.09	1002	1004	3.878
2	4-Thujanol	1.20	1065	1070	4.05
3	D-Limonene	57.77	1031	1028	4.275
4	γ-Terpinene	11.29	1057	1059	4.55
5	Terpinolene	5.50	1086	1089	4.76
6	Fenchol	0.38	1123	1121	5.083
7	1-Terpineol	0.61	1136	1140	5.206
8	β-Terpineol	0.50	1151	1153	5.358
9	Terpinen-4-ol	0.43	1176	1180	5.75
10	α-Terpineol	3.93	1186	1190	5.965
11	α-Terpinyl acetate	0.73	1330	1328	5.999
12	Citral	0.51	1245	1242	6.803
13	Neryl Acetate	0.70	1360	1362	8.531
14	α-Bergamotene	1.67	1435	1433	9.707
15	α-Himachalene	2.78	1452	1448	11.157
16	δ-Tocopherol	0.54	2951	2953	35.945
17	Farnesol	5.44	1709	1711	36.587
18	γ-Tocopherol	0.84	3051	3055	37.346
19	α-Tocopherol	0.37	3110	3112	38.345
	Total (%)	98.28			

**Table A2:** Chemical components of CSEO by GC-MS analysis.

No.	Compound	Content (%)	RI		RT (min)
			Measured	Documented	
1	3-Carene	0.5	1007	1009	3.976
2	D-Limonene	35.06	1031	1028	4.163
3	Isopulegol	0.18	1151	1146	4.55
4	Dihydrocarveol	0.4	1187	1190	4.314
5	Linalool	4.04	1100	1101	4.799
6	p-Mentha-2,8-dienol	11.61	1081	1078	5.216
7	5-Caranol	0.53	1281	1286	5.323
8	Chrysanthenol	0.9	1162	1164	5.377
9	Carveol	12.44	1204	1200	5.745
10	Pseudocarveol	0.77	1191	1186	5.877
11	Thujyl Alcohol	5.55	1148	1146	5.617
12	Isopiperitenol	1.72	1754	1752	5.745
13	Perillyl Alcohol	0.35	1291	1294	6.097
14	D-Carvone	3.22	1241	1245	6.666
15	Citral	1.71	1245	1242	6.857
16	Perillal	0.79	1274	1276	7.101
17	Cyclohexanol	1.18	851	849	7.273
18	α-Copaene	0.56	1374	1372	8.728
19	β-Copaene	1.13	1421	1424	8.919
20	Valencene	2.1	1474	1479	10.941
21	δ-Cadinene	0.73	1483	1486	11.372
22	Spathulenol	0.42	1576	1578	8.326
23	Sesquibenihiol	0.84	1786	1788	8.458
24	α-terpinene	0.19	1014	1016	19.963
25	Elaidic Acid Methyl Ester	0.72	2081	2084	22.823
26	Tangeretin	2.6	3196	3198	38.781
27	Stigmasterol	0.54	3174	3170	39.893
	Total (%)	90.78			

**Table A3:** Chemical components of CLEO by GC-MS analysis.

No.	Compound	Content (%)	RI		RT (min)
			Measured	Documented	
1	Pseudolimonene	0.48	1002	1004	3.952
2	Terpilene	3.1	1019	1016	4.04
3	D-Limonene	21.74	1031	1028	4.099
4	γ-Terpinene	3.28	1057	1059	4.182
5	α-Fenchene	30.19	943	947	4.261
6	Isoterpinene	5.39	1057	1083	4.437
7	Linalyl Formate	15.46	1216	1219	4.53
8	Fenchol	1.86	1123	1121	4.711
9	Chrysanthenol	1.68	1162	1164	4.902
10	α-Terpineol	3.07	1186	1190	5.23
11	Verbenol	0.57	1152	1145	5.402
12	Citral	3	1245	1242	5.617
13	Neryl Acetate	1.02	1360	1362	6.024
14	Myrac Aldehyde	0.94	1506	1509	6.778
	Total (%)	91.78			

**Table A4:** Chemical components of BCSEO by GC-MS analysis.

No.	Compound	Content (%)	RI		RT (min)
			Measured	Documented	
1	3-Carene	0.78	1007	1009	3.976
2	D-Limonene	34.6	1031	1028	4.192
3	Linalool	7.07	1100	1101	4.809
4	trans-Mentha-2,8-dien-1-ol	1.29	1126	1123	5.078
5	trans-p-Mentha-2,8-dienol	0.56	1075	1078	5.186
6	p-Mentha-2,8-dien-1-ol	1.47	1137	1134	5.23
7	Neoisopulegol	0.78	1141	1144	5.304
8	α-Terpineol	6.97	1186	1190	5.999
9	Carveol	4.55	1204	1200	6.034
10	(+)-3-Carene	0.5	991	995	6.504
11	Linalyl acetate	0.77	1249	1251	6.558
12	Carvone	1.07	1247	1243	6.602
13	Citral	2.21	1245	1242	6.856
14	Perillal	0.84	1274	1276	7.082
15	Estragole	0.48	1196	1200	7.165
16	p-Mentha-1(7),8(10)-dien-9-ol	0.85	1281	1287	7.243
17	Isoanethole	0.64	1195	1199	7.395
18	α-Copaene	0.98	1374	1372	8.722
19	Cyclododecanol	1.64	1574	1575	9.173
20	β-Guaiene	0.62	1486	1490	10.921
21	2,5-Di-Tert-Butylphenol	1.51	1509	1514	10.991
22	δ-Cadinene	1.85	1483	1486	11.382
23	γ-himachalene	0.78	1486	1482	12.621
24	β-Costol	0.45	1774	1778	12.753
25	β-Sinensal	1.55	1690	1694	14.908
26	α-Sinensal	1.2	1746	1748	16.04
27	Docosahexaenoic acid methyl ester	0.43	2410	2413	17.21
28	Hexadecanoic acid, methyl ester	0.94	1925	1927	19.522
29	Elaidic Acid Methyl Ester	3.52	2081	2084	22.852
30	Methyl Lineoleate	0.42	2088	2092	24.4
31	Squalene Oxide	0.48	2695	2693	34.206
32	Tangeretin	5.79	3196	3198	38.825
33	Scutellarein Tetramethyl Ether	2.26	3183	3186	38.879
34	Stigmasterol	0.92	3174	3170	39.893
	Total (%)	90.77			

**Table A5:** Chemical components of CBEO by GC-MS analysis.

No.	Compound	Content (%)	RI		RT (min)
			Measured	Documented	
1	Isomenthone	10.62	1174	1170	5.485
2	α-Terpineol	1.3	1186	1190	5.642
3	Citronellol	29.56	1241	1237	5.955
4	Nerol	9.02	1258	1254	6.455
5	3,7-Dimethyloct-6-enyl ethyl carbonate	5.99	1517	1514	6.744
6	Myrtanol	0.41	1253	1257	6.93
7	Geranyl Ethyl Carbonate	0.99	1549	1553	7.038
8	Guaia-6,9-Diene	11.52	1443	1447	7.268
9	3-Carene	0.35	1007	1009	8.056
10	α-Copaene	0.92	1374	1372	8.169
11	β-Bourbonene	2.83	1381	1384	8.556
12	α-Guaiene	1.05	1440	1442	8.909
13	γ-himachalene	3.85	1486	1482	9.58
14	α-Muurolene	1.77	1468	1469	9.966
15	β-copaene	0.41	1427	1424	10.03
16	Ledene	2.58	1497	1495	10.099
17	γ-Cadinene	0.45	1517	1513	10.26
18	T-Cadinol	3.65	1645	1640	10.334
19	Calamenene	1.47	1518	1522	10.495
20	Neryl Isobutyrate	1.85	1514	1511	10.633
21	Sesquibenihiol	0.78	1786	1788	10.721
22	Dehydrosaussurea Lactone	0.36	1836	1838	11.005
23	Geranyl tiglate	2.3	1699	1703	11.313
	Total (%)	94.03			
